# Diet characterisation of solitary bees on farmland: dietary specialisation predicts rarity

**DOI:** 10.1007/s10531-016-1191-x

**Published:** 2016-08-20

**Authors:** T. J. Wood, J. M. Holland, D. Goulson

**Affiliations:** 1 School of Life Sciences, The University of Sussex, Falmer, East Sussex BN1 9QG UK; 2 The Game and Wildlife Conservation Trust, Burgate Manor, Fordingbridge, Hampshire SP6 1EF UK

**Keywords:** Wild bees, Pollinators, Agri-environment, Host plants, Agro-ecosystems, Bee conservation, Pollen diet

## Abstract

Changes in agricultural practice across Europe and North America have been associated with declines in wild bee populations. Bee diet breadth has been associated with sensitivity to agricultural intensification, but much of this analysis has been conducted at the categorical level of generalist or specialist, and it is not clear to what extent the level of generalisation within generalist species is also associated with species persistence. We used pollen load analysis to quantify the pollen diets of wild solitary bees on 19 farms across southern England, UK. A total of 72 species of solitary bees were recorded, but only 31 species were abundant enough to allow for formal diet characterisation. The results broadly conformed to existing literature with the majority of species polylectic and collecting pollen from a wide range of plants. Pollen load analysis consistently identified pollens from more plant species and families from each bee species than direct observation of their foraging behaviour. After rarefaction to standardise pollen load sample sizes, diet breadth was significantly associated with frequency of occurrence, with more generalist bees present on more farms than less generalist bees. Our results show that the majority of bee species present on farmland in reasonable numbers are widely variable in their pollen choices, but that those with the broadest diet were present on the greatest number of farms. Increasing the diversity of plants included in agri-environment schemes may be necessary to provide a wider range of pollen resources in order to support a diverse bee community on farmland.

**Electronic supplementary material:**

The online version of this article (doi:10.1007/s10531-016-1191-x) contains supplementary material, which is available to authorized users.

## Introduction

Since the Second World War, many wild bee species have undergone substantial range contractions and extinctions across Europe and North America (Biesmeijer et al. 2006; Kosior et al. 2007; Goulson et al. 2008; Bartomeus et al. 2013). These declines have been linked to changes in agricultural practice which have reduced the abundance and diversity of flowering plants, reducing the amount and range of food resources available to foraging bees (Ollerton et al. 2014; Scheper et al. 2014; Goulson et al. 2015). A loss of bees from agricultural areas is of concern because of their important role as pollinators of both wild and crop plants (Ollerton et al. 2011; Garibaldi et al. 2013).

Partly to address these declines in wild bees, agri-environment schemes have been implemented across the European Union as part of the Common Agricultural Policy. Designed to deal with more general declines in agricultural biodiversity, they offer an opportunity to provide important foraging resources for wild bees. However, there are still important knowledge gaps relating to the conservation of wild bees, in part relating to an incomplete understanding of their agro-ecology (Dicks et al. 2013). Previous authors have found that bee diet breadth is associated with sensitivity to habitat loss and agricultural intensification, with generalists faring better than specialists (Bommarco et al. 2010; De Palma et al. 2015). However, much of this analysis has been conducted at the categorical level of generalist or specialist, and it is not clear to what extent the level of generalisation within generalist species is also associated with species persistence. Providing appropriate foraging resources for wild bees on farmland is important if their populations are to be maintained, but the lack of data on their contemporary diets is detrimental as the assessment of the efficacy of current agri-environment schemes often has to work with an incomplete knowledge of bee species requirements (Dicks et al. 2015).

Whilst there has been reasonable assessment of the diet of bumblebees on and off agricultural land (Goulson et al. 2005; Carvell et al. 2006, 2007; Kleijn and Raemaekers 2008), less is known about the diet of the wider bee community (though see Scheper et al. 2014). In terms of the number of species, the bee fauna in temperate regions is dominated by ‘solitary’ bees that live independently and collect pollen to provision their own offspring. This term is used generically to contrast this group against the social bumblebees and the honeybee *Apis mellifera*. However, the ‘solitary’ bees include many species within the Halictidae that show variably expressed eusocial behaviour (Plateaux-Quénu 2008; Davison and Field 2016). Consequently, whilst not technically correct, the term solitary bees is used from here on into mean any non-parasitic (i.e. they forage for their own pollen), non-corbiculate (i.e. non-*Bombus*, non-*Apis*) bee species.

Some field studies have used direct field observations of pollen foraging behaviour to identifying pollen preferences in wild bees (e.g. Minckley et al. 1999; Steffan-Dewenter and Tschantke 2001; Goulson et al. 2005). However, many wild bees are not flower constant and do not exclusively use the same flower species during a foraging trip, instead visiting and carrying pollen from many different flowering plants at once (Westrich 1989; Beil et al. 2008). Additionally, wild bees can forage over considerable distances (Beil et al. 2008; Zurbuchen et al. 2010) and may visit flowers inaccessible to or outside the areas visited by recorders. As a result, direct observations may not capture the full range of plant species visited for pollen, and may not accurately measure the relative contribution that different host plants make to the overall diet as the proportion of pollen collected from each particular plant species is unknown. In establishing more definitive pollen preferences and diet breadth ranges, microscopic analysis of pollen loads has been favoured as the total volume of pollen collected from different sources can be quantified. The level of specialisation, as either oligolectic (collecting pollen from one plant family or genus) or polylectic (collecting pollen from many plant families) can then be made with greater confidence (Westrich 1989; Müller 1996; Müller and Kuhlmann 2008). The use of pollen load analysis is becoming more widely used in field studies to assess pollen utilisation of both semi-natural and agri-environment scheme plants (Carvell et al. 2006; Kremen and Williams 2007; Beil et al. 2008) and to quantify historical bee diets through the analysis of remaining pollen loads present on museum specimens (Kleijn and Raemaekers 2008; Scheper et al. 2014).

In this study we characterise the pollen foraging diets of wild solitary bees on farmland in southern England, UK using pollen load analysis. Within a British context this is the most comprehensive assessment of solitary bee species since Chambers (1968). We examine the diet breadth results in the context of frequency of occurrence, with the prediction that those species with a wider diet will be present on a greater number of farms. This information will be of use to land managers and policy makers interested in maintaining pollinator populations on agricultural land.

## Methods

### Study area

In England, two tiers of environmental stewardship were established in 2005. Entry Level Stewardship (ELS, Natural England 2013a) was open to all farmers. Higher Level Stewardship (HLS, Natural England 2013b) which was targeted to high priority areas provided greater financial rewards for more substantial and rigorous agri-environmental schemes. At the time of the study, around 70 % of farms in England were in some form of environmental stewardship (JNCC Joint Nature Conservation Committee 2014). Nineteen farms were selected in Hampshire and West Sussex, UK. Nine farms were under HLS management and were implementing pollinator-friendly management. This consisted of an average of 5.6 ± 0.1 ha of flower-rich grassland per farm, typically established by sowing areas with seed mixes containing c.15–30 flowering forb species (Carvell et al. 2007). Ten farms were under ELS and were not implementing any pollinator-friendly management. ELS management can include schemes targeted at pollinators that can benefit wild bee populations (Pywell et al. 2012), but the overall uptake of such schemes within ELS is low (Elliot et al. 2010). Consequently, general ELS farms not implementing any specifically pollinator-friendly management were selected as the control group for this study. The floral communities on the studied farms consequently consisted of sown plants in conservation areas and wild plants persisting in the wider farmed environment. Farms were predominantly arable, or mixed arable/dairy with wheat, barley, oilseed rape and permanent/silage grassland as the major crops representing the dominant agricultural practices in this region.

### Bee surveys and sample collection

In 2013 and 2014, a standardised 3 km transect was designed for each farm, passing through all major habitat types present, excluding crops. These habitats types can be divided into flower-rich pollinator-focused schemes, non-agricultural grassland and hedgerows and woodland edges. Details of habitat types and crops for each farm can be found in Appendix I in supplementary material. Transects on HLS farms were designed to survey as many pollinator-focused schemes as possible and passed through an average of 1496 ± 148 m of flower-rich habitat in an average of 3.8 ± 0.2 discrete habitat patches. Solitary bee activity was recorded along the transect following standard bee walk methodology (Carvell et al. 2007), with all solitary bees within 2 m of the recorder identified to species level. Individuals that could not be named in the field were netted for later identification. The first flowering plant species visited and the purpose of the visit, for either pollen or nectar, was recorded. *Hylaeus* species, which lack scopal (pollen collecting) hairs on their body, instead ingesting pollen and regurgitating it in the nest, cannot be reliably determined to be foraging for pollen and so all plant visits were recorded simply as visits. Sixteen farms (eight HLS, eight ELS) were surveyed in 2013. Transects were walked three times through the season, between 25th May and 5th June, 26th June–15th July and 3rd–11th August 2013. Seventeen farms (eight HLS, nine ELS) were surveyed in in 2014. Transects were walked three times through the season, between 17th and 27th May, 21st June–9th July and 3rd–15th August 2014.

In 2015 farms were surveyed on time based rather than distance based transects. ELS farms were surveyed for 3 h with 1.5 h on non-agricultural grass habitats and 1.5 h on woody hedgerow/woodland edge habitats. HLS farms were surveyed for 3 h with 1 h on pollinator-focused flower-rich schemes, 1 h on non-agricultural grass habitats and 1 h on woody hedgerow/woodland edge habitats. The survey followed standard bee walk methodology as described above, but at a reduced pace to ensure thorough sampling. In addition, female bees with clearly visible pollen on their body were collected, placed in individual Eppendorf tubes and frozen. Samples of all flowering plant species present were collected to form a pollen reference library. Fourteen farms (7 HLS, 7 ELS) were surveyed in 2015. Transects were walked four times throughout the season, between 22 April and 13th May, 26th May and 17th June, 25th June–4th July and 29th July–10th August 2015. All bee surveys were conducted between 0930 and 1700 h when the temperature was above 13 °C with at least 60 % clear sky, or above 17 °C with any level of cloud. No surveys were conducted when it was raining. All bee surveys were conducted by the same individual (TJW) to minimise recorder bias.

### Pollen identification

The scopal pollen load of foraging bees collected in 2015 was analysed by light microscopy using the method outlined by Westrich and Schmidt (1986). Before removing pollen from the scopae, the total load was estimated relative to a full load for that species, ranging from 8/8 (full load) to 1/8 (one eighth load). The pollen grains were removed from the scopae using an entomological pin and transferred to a drop of water on a microscope slide. Pollen that was not clearly held in the scopae was not sampled as this may have become attached to other parts of the body during nectar visits to non-host plant flowers. The slide was gently heated to allow grains to absorb water and achieve their maximum size and to evaporate excess water. Molten glycerine jelly stained with fuchsin was then added and the slide was sealed with a coverslip. The proportion of the load comprised of different plant species was estimated along three randomly selected lines across the cover slip at a magnification of ×400. The proportion of the load by volume was estimated by the relative area of the slide occupied by each plant species, rather than the absolute number of grains, in order to better reflect the total volume of pollen collected, an important correction in mixed loads where pollen grains of different plant species often differ widely in size (Cane and Sipes 2006). Species representing less than 1 % of the load were excluded from further analysis as they may have arisen from contamination (Westrich and Schmidt 1986). As specimens were individually netted and stored in clean Eppendorf tubes such contamination was expected to be minimal.

The proportions of pollen collected were corrected according to the overall size of each load to give a final weight, e.g. a full load (8/8) comprised of 50 % *Centaurea nigra* and 50 % *Leucanthemum vulgare* would receive a final *C. nigra* weight of 50 and a final *L. vulgare* weight of 50, whereas a quarter load (2/8) comprised of 100 % *Hypochaeris radicata* would receive a final *H. radicata* weight of 25. The pollen grains were identified to species using Sawyer (1981) and the reference collection assembled during the project. Where identification to species level was not possible, pollen was identified to genus, for example in *Brassica*, *Plantago* and *Geranium*.

### Diet characterisation

Characterisation of floral preferences in pollen host plants was carried out for species with a minimum of three pollen load samples following Müller and Kuhlmann (2008). A small sample size may result in mischaracterisation of bee diets as certain plant families may be under or overrepresented. However, in all cases for bees with sample sizes of less than five the results conformed closely to more extensive previous studies (see Results). Consequently, for species with small sample sizes the results presented here should be viewed within this broader context. In characterising diet we used the categories laid out by Müller and Kuhlmann (2008) who modified the work of Cane and Sipes (2006) to include additional subcategories of oligolecty and polylecty (Appendix II in supplementary material). This modification added the category ‘polylectic with a strong preference’ as this pattern of host use exists in many species (Müller 1996). Müller and Kuhlmann (2008) used two approaches to characterise oligolecty for a given bee species using microscopic analysis of pollen loads. The first averages use over all individuals so a bee species is deemed oligolectic if 95 % of the pollen grains (or 95 % of the pollen by volume) is from one plant family or genus (Müller 1996). The second approach looks at the proportion of pure and mixed pollen loads so a bee species is deemed oligolectic if 90 % of females collect pure pollen loads of one plant family or genus (Sipes and Tepedino 2005). These criteria are summarised in Appendix III in supplementary material. Both methods produce similar results (Müller and Kuhlmann 2008), but in the few cases where they produced different answers the category with the lower degree of specialisation was used in our analysis.

Flower visit observations from the 2013, 2014 and 2015 transects were pooled. Differences in the number of plant species and families utilised for pollen detected by the direct observation and pollen load analysis techniques were tested using Mann–Whitney U tests. When comparing diet breadth between different bee species, rarefaction must be used to reduce the impact of differing samples sizes between species and the consequent effect on diet breadth calculations (Williams 2005). A rarefaction procedure was used to calculate the number of pollen types from different plant species (pollens) each different bee species would be expected to collect for a standardised number of pollen loads. Here we rarefied the diets of bees for (i) species with a minimum of ten pollen loads and (ii) species with a minimum of three pollen loads. A subsample of (i) 12 pollen loads (smallest sample size over 10, *Andrena subopaca* see Table 1) and (ii) three pollen loads is made from the observed frequency of pollens collected, chosen at random without replacement 1000 times. As this procedure is designed for use on integer data, the pollen load data was first transformed. For example, with a sample size of 14, the percentage of pollen collected from each plant species was multiplied by the sample size to give a whole pollen load equivalent, e.g. 40 % becomes 5.6 pollen loads. These values were all multiplied by ten and rounded to the nearest whole number to give an integer equivalent that was used in the rarefaction procedure. For the group with a minimum sample size of ten pollen loads the relationship between diet breadth (the number of pollens collected) and frequency of occurrence (the number of farms at which the bee species was recorded at least once over the 3 year survey period) was tested using a linear model with a Gaussian distribution as the response variable conformed to a normal distribution. Bee family was included in the model as a fixed factor to control for the possible impact of bee phylogeny on the results. For the group with a minimum sample size of three pollen loads the response variable could not be transformed to normality, and for this dataset the relationship between diet breadth and frequency of occurrence was tested using Spearman’s rank correlation.

**Table 1:** Host plant spectrum and inferred category of host use in sampled farmland solitary bee species

Bee species	*n*	*N*	Results of microscopic analysis of pollen grains (% pollen grains)	% Pure loads of preferred host	% Loads with preferred host	Host range	Host range in the literature
*Andrena alfkenella*	6	3	API 97.0, other 3.0	33.3	100.0	Broadly oligolectic (Apiaceae)^a^	Polylectic
*Andrena bicolor*	16	7	AST 29.6, BRA 21.4, CUC 13.7, API 13.7, CAM 10.5, LIL 7.8, other 2.0	37.5	37.5	Polylectic s.s.	Polylectic
*Andrena chrysosceles*	32	9	API 45.6, BRA 32.1, ROS 14.9, MAL 3.1, AST 2.2, other 2.1	43.8	78.1	Polylectic s.s.	Polylectic
*Andrena cineraria*	9	3	BRA 53.8, API 27.9, ROS 12.9, RHA 3.2, other 2.2	33.3	77.8	Polylectic s.s.	Polylectic
*Andrena dorsata*	22	9	ROS 66.6, API 11.8, BRA 9.7, AST 7.3, FAB 4.7	50.0	68.2	Polylectic s.s.	Polylectic
*Andrena flavipes*	45	10	AST 56.0, BRA 20.1, FAB 10.5, ROS 5.1, API 5.0, other 3.3	15.6	86.7	Polylectic s.s.	Polylectic
*Andrena florea*	3	2	CUC 100.0	100.0	100.0	Narroly oligolectic (*Bryonia dioica*)	Narrowly oligolectic (*Bryonia*)
*Andrena haemorrhoa*	40	12	ROS 37.8, BRA 31.5, AST 14.7, RES 6.3, CAP 4.9, other 3.0	10.0	45.0	Polylectic s.s.	Polylectic
*Andrena labiata*	3	1	CAR 50.0, VER 16.7, RAN 12.5, BRA 10.8, AST 7.5, GER 2.5	0.0	66.7	Polylectic s.s.	Polylectic
*Andrena minutula*	15	8	API 56.4, BRA 23.6, ROS 11.6, AST 8.0, other 0.4	20.0	60.0	Polylectic s.s.	Polylectic
*Andrena minutuloides*	8	2	API 100.0	100.0	100.0	Broadly oligolectic (Apiaceae)^a^	Polylectic
*Andrena nigroaenea*	16	6	API 39.4, AST 34.5, BRA 14.2, RAN 6.3, RES 3.1, other 2.5	18.8	62.5	Polylectic s.s.	Polylectic
*Andrena nitida*	23	10	BRA 45.6, API 16.3, ACE 9.9, RHA 9.1, AST 4.7, SOL 3.8, LAM 3.8, ROS 2.4, other 4.5	21.7	69.6	Polylectic s.s.	Polylectic
*Andrena scotica*	18	7	BRA 65.7, ACE 19.6, API 5.4, ROS 4.2, other 5.1	55.6	77.8	Polylectic s.s.	Polylectic
*Andrena semilaevis*	97	9	API 91.3, BRA 5.3, VER 1.9, other 1.5	75.3	97.9	Polylectic with a strong preference (Apiaceae)	Polylectic
*Andrena subopaca*	12	4	API 48.4, BRA 19.3, ROS 18.2, VER 9.2, FAB 2.6, other 2.3	33.3	50.0	Polylectic s.s.	Polylectic
*Halictus tumulorum*	21	10	AST 24.2, RAN 22.2, FAB 16.9, ROS 13.9, BRA 12.4, RUB 6.82, LIL 3.0, other 2.62	38.1	71.4	Polylectic s.s.	Polylectic
*Lasioglossum albipes*	3	2	RAN 65.3, ROS 33.3, other 1.3	33.3	66.7	Polylectic s.s.	Polylectic
*Lasioglossum calceatum*	38	11	AST 46.9, BRA 17.5, ROS 11.3, API 6.0, LAM 3.9, BER 3.7, OLE 3.5, RAN 2.6, other 4.6	42.1	68.4	Polylectic s.s.	Polylectic
*Lasioglossum fulvicorne*	7	2	BRA 67.2, ROS 26.9, API 5.0, other 0.8	57.1	71.4	Polylectic s.s.	Polylectic
*Lasioglossum lativentre*	9	3	FAB 95.3, RAN 2.6, AST 2.1	77.8	88.9	Polylectic with a strong preference (Fabaceae)	Polylectic
*Lasioglossum leucopus*	7	6	BRA 68.1, API 22.9, RAN 6.3, AST 2.5, other 0.3	0.0	57.1	Polylectic s.s.	Polylectic
*Lasioglossum leucozonium*	21	6	AST 95.3, RAN 4.7	71.4	100.0	Polylectic with a strong preference (Asteraceae)	Polylectic
*Lasioglossum malachurum*	437	12	AST 73.1, BRA 9.9, ROS 3.3, other 13.7	53.3	83.3	Polylectic with a strong preference (Asteraceae)	Polylectic
*Lasioglossum morio*	7	5	BRA 51.2, AST 15.1, API 12.1, CAP 11.2, ROS 8.4, other 2.1	28.6	42.9	Polylectic s.s.	Polylectic
*Lasioglossum parvulum*	7	5	ROS 28.5, LAM 23.5, RAN 20.2, API 11.2, ACE 9.4, AST 5.9, other 1.3	0.0	42.9	Polylectic s.s.	Polylectic
*Lasioglossum pauxillum*	70	10	AST 62.1, API 9.8, RAN 9.4, ROS 8.7, BRA 3.5, VER 2.3, FAB 2.2, other 2.0	45.7	68.6	Polylectic s.s.	Polylectic
*Lasioglossum villosulum*	25	5	AST 100.0	100.0	100.0	Broadly oligolectic (Asteraceae)	Polylectic
*Lasioglossum xanthopus*	7	3	AST 67.8, BRA 32.0, other 0.2	42.9	85.7	Mesolectic	Polylectic
*Lasioglossum zonulum*	4	1	ROS 53.6, AST 21.6, CAR 12.9, RAN 6.1, CAP 4.3, other 1.4	0.0	100.0	Polylectic s.s.	Polylectic
*Melitta tricincta*	3	1	ORO 100.0	100.0	100.0	Narrowly oligolectic (*Odontites vernus*)	Narrowly oligolectic (*Odontites*)

Only species with a minimum of three collected pollen loads are included. *n* total number of pollen loads, *N* number of pollen loads from different localities, *s.s.* sensu strictu, *Plant taxa ACE*, Aceraceae, *API* Apiaceae, *AST* Asteraceae, *BER* Berberidaceae, *BRA* Brassicaceae, *CAM* Campanulaceae, *CAP* Caprifoliaceae, *CAR* Caryophyllaceae, *CUC* Cucurbitaceae, *FAB* Fabaceae, *GER* Geraniaceae, *LAM* Lamiaceae, *LIL* Lilaceae, *MAL* Malvaceae, *OLE* Olaceae, *ORO* Orobanchaceae, *RES* Resedaceae, *RAN* Ranunculaceae, *RHA* Rhamnaceae, *ROS* Rosaceae, *RUB* Rubiaceae, *SOL* Solanaceae, *VER* Veronicaceae

^a^Pollen data only available for summer generation females

Additionally, we also investigated the impact of farm type (HLS or ELS) on diet breadth and frequency of occurrence to ensure that the effect was consistent across different management types. Fourteen bee species had a minimum of ten pollen loads from either HLS or ELS farms (nine species had a minimum of ten pollen loads from both farm types, five species has a minimum of ten pollen loads from only one farm type). Following the same protocol these data were rarefied to a sample size of ten pollen loads and were tested in a linear model with a Gaussian distribution with diet breadth and farm management type included as fixed factors. All statistical analyses were conducted in R version 3.1.1 (R Development Core Team 2016) using the package vegan (Oksanen et al. 2015) to calculate diet rarefaction scores.

## Results

A total of 72 species of solitary bee was recorded over the sampling period (full list in Appendix IV in supplementary material). Fifty-six solitary bee species were observed making 1416 pollen foraging trips to 62 flowering plant species from 19 families. One thousand and fifty-four bees with pollen loads from 47 solitary bee species were collected for microscopic pollen load analysis. Pollen analysis detected 93 pollen types from 32 plant families. Thirty-one solitary bee species were collected in sufficient quantities to allow diet breadth characterisation (Table 1).

The host plant use results broadly conformed to existing literature (Chambers 1968; Westrich 1989; Amiet et al. 2010), with the majority of species characterised as polylectic or polylectic with some preferences. Five species (*Andrena alfkenella*, *A. florea*, *A. minutuloides*, *Lasioglossum villosulum* and *Melitta tricincta*) were characterised as oligolectic. However, *A. alfkenella*, *A. minutuloides* and *L. villosulum* are not considered oligolectic by other authors (see Discussion). Excluding these three doubtful species a total of 15 solitary bee species well known to be oligolectic was recorded (Table 2). Only *A. florea* and *M. tricincta* were collected in sufficient numbers to allow formal diet characterisation. For the other 13 oligolectic species, observed pollen visits were in line with their expected host preferences. Important pollen sources in the study region are summarised in Table 2, with likely pollen host plants based on observed nectar visits to suitable plants present at the localities where they were recorded.

**Table 2:** Oligolectic solitary bee species recorded during the survey and their observed pollen host plants in the study area

Bee species	Pollen sources in the study area	Host range in the literature
*Andrena florea*	*Bryonia dioica*	Narrowly oligolectic (*Bryonia*)
*Andrena fulvago*	none recorded (likely *Hypochaeris radicata*)	Broadly oligolectic (Asteraceae)
*Andrena humilis*	*Hypochaeris radicata*	Broadly oligolectic (Asteraceae)
*Andrena nitidiuscula*	none recorded (likely *Heracleum sphondylium,* ***Daucus carota***)	Broadly oligolectic (Apiaceae)
*Andrena wilkella*	*Trifolium repens,* ***Trifolium hybridum***, ***Lotus corniculatus***	Broadly oligolectic (Fabaceae)
*Anthophora furcata*	*Stachys sylvatica*	Broadly oligolectic (Lamiaceae)
*Chelostoma campanularum*	none recorded (likely *Campanula trachelium*)	Narrowly oligolectic (*Campanula*)
*Chelostoma florisomne*	*Ranunculus repens*	Narrowly oligolectic (*Ranunculus*)
*Colletes daviesanus*	***Achillea millefolium*** *, Tripleurospermum inodorum,* ***Leucanthemum vulgare***	Broadly oligolectic (Asteraceae)
*Hylaeus signatus*	*Reseda lutea*	Narrowly oligolectic (*Reseda*)
*Melitta leporina*	*Trifolium repens*	Broadly oligolectic (Fabaceae)
*Melitta tricincta*	*Odontites vernus*	Narrowly oligolectic (*Odontites*)
*Osmia leaiana*	***Centaurea nigra*** *, Crepis capillaris*	Broadly oligolectic (Asteraceae)
*Osmia spinulosa*	***Achillea millefolium*** *, Helminthotheca echioides*	Broadly oligolectic (Asteraceae)
*Panurgus calcaratus*	none recorded (likely *Hypochaeris radicata*)	Broadly oligolectic (Asteraceae)

Likely pollen sources are based on nectar visits to suitable host plants present at the locality

Plants sown as part of pollinator-friendly management are highlighted in bold

**Fig. 1:**
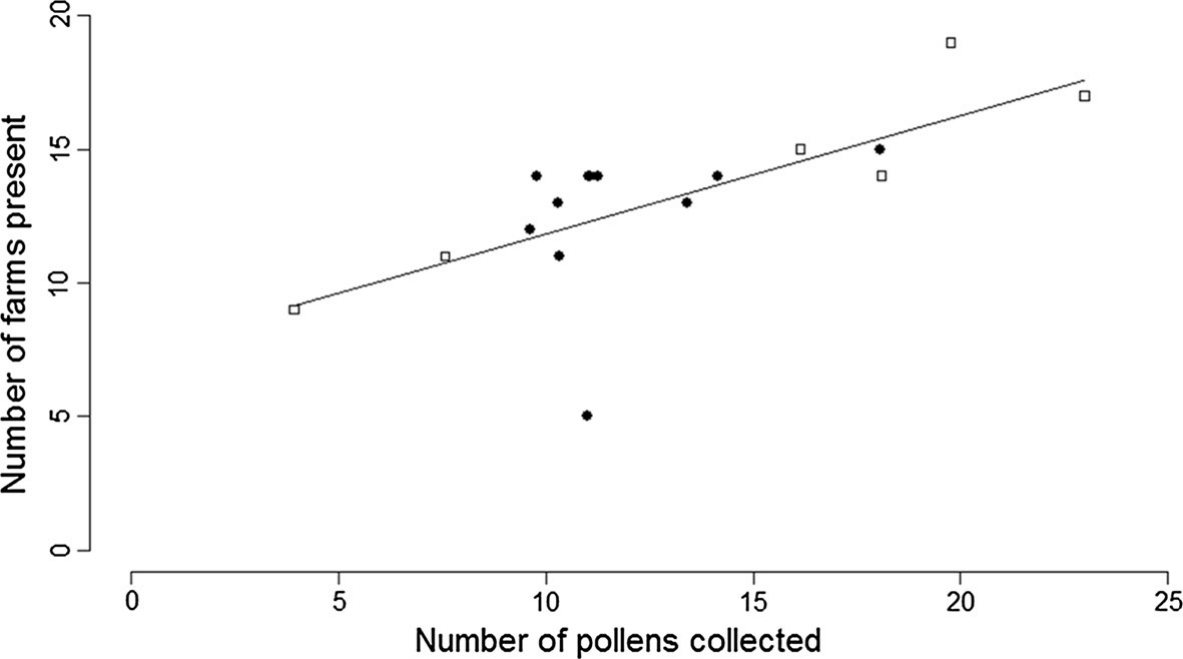
The relationship between diet breadth of solitary bee species (n = 17) after rarefaction (to a standardised sample size of 12 pollen loads) and the number of farms each bee species was recorded on. *Circles* bee species from the family Andrenidae, *Squares* bee species from the family Halictidae

Excluding the narrowly oligolectic *A. florea* and *M. tricincta* (that each only collect pollen from one plant species in Britain), direct observation recorded bees collecting pollen from an average of 6.9 ± 0.9 plant species from an average of 3.4 ± 0.3 plant families per bee species (Table 3). Microscopic pollen analysis detected significantly more pollens from an average of 13.6 ± 1.9 plant species from an average of 7.6 ± 0.9 plant families per bee species (W = 211.5, p = 0.001; W = 151.5, p < 0.001 respectively). Of the 13 additional plant families detected in pollen load analysis, seven were represented by woody genera in the study area, specifically Aceraceae (*Acer*), Aquifoliaceae (*Ilex*), Berberidaceae (*Berberis*), Cornaceae (*Cornus*), Fagaceae (*Castanea*, *Fagus*), Malvaceae (*Tilia*) and Rhamnaceae (*Rhamnus*). For the 17 species with a minimum sample size of 10 analysed pollen loads, after rarefaction there was a significant relationship between diet breadth and frequency of occurrence (Fig. 1, t_14,16_ = 3.411, p = 0.004, adjusted R^2^ = 0.413). There was no impact of bee family on this relationship (t_14,16_ = 0.186, p = 0.855). After removing bee family from the model diet breadth was still a significant predictor of frequency of occurrence (t_15,16_ = 3.756, p = 0.002, adjusted R^2^ = 0.450). The same relationship was found if the analysis is repeated for all 31 species with a minimum of 3 analysed pollen loads (Spearman’s rho = 0.794, p < 0.001). There was no impact of farm management type on the relationship between diet breadth and frequency of occurrence (t_20,22_ = 0.616, p = 0.545) with diet breadth remaining significant with (t_20,22_ = 2.384, p = 0.027, adjusted R^2^ = 0.150) and without (t_21,22_ = 2.379, p = 0.027, R^2^ = 0.175) the inclusion of this term in the model.

**Table 3:** Number of pollens from different flowering plant species and flowering plant families collected by solitary bee species (excluding narrowly oligolectic species), by direct observation and pollen load analysis

Bee species	*obs*	*n*	Number of plant species		Rarefied	Number of plant families	
			Observations	Pollen loads		Observations	Pollen loads
*Andrena alfkenella*	10	6	2	7		1	5
*Andrena bicolor*	17	16	5	11	9.61	4	9
*Andrena chrysosceles*	38	32	7	16	13.40	4	8
*Andrena cineraria*	16	9	6	9		3	6
*Andrena dorsata*	34	22	9	15	10.30	5	5
*Andrena flavipes*	70	45	14	28	18.06	4	12
*Andrena haemorrhoa*	43	40	6	18	11.03	5	11
*Andrena labiata*	2	3	1	6		1	6
*Andrena minutula*	30	15	9	11	9.78	3	5
*Andrena minutuloides*	10	8	2	2		1	1
*Andrena nigroaenea*	13	16	5	16	11.07	4	10
*Andrena nitida*	24	23	7	16	14.14	5	12
*Andrena scotica*	13	18	4	12	11.24	3	9
*Andrena semilaevis*	127	97	7	18	10.33	3	9
*Andrena subopaca*	7	12	3	13	11.00	3	8
*Halictus tumulorum*	23	21	14	19	16.14	5	11
*Lasioglossum albipes*	3	3	2	4		2	4
*Lasioglossum calceatum*	70	38	17	31	19.76	7	16
*Lasioglossum fulvicorne*	6	7	3	4		3	4
*Lasioglossum lativentre*	8	9	3	5		2	3
*Lasioglossum leucopus*	5	7	2	8		2	5
*Lasioglossum leucozonium*	51	21	9	8	7.56	2	2
*Lasioglossum malachurum*	553	437	22	50	22.98	8	22
*Lasioglossum morio*	6	7	6	11		5	7
*Lasioglossum parvulum*	6	7	4	9		4	7
*Lasioglossum pauxillum*	93	70	17	28	18.09	4	12
*Lasioglossum villosulum*	32	25	7	5	3.93	1	1
*Lasioglossum xanthopus*	8	7	1	5		1	4
*Lasioglossum zonulum*	7	4	5	9		4	6
Average			6.9 ± 1.0	13.6 ± 1.9	12.9 ± 1.2	3.4 ± 0.3	7.6 ± 0.9

Data was rarefied for species with a mininum of ten pollen loads

*obs* number of observations, *n* number of pollen loads

## Discussion

Amongst the solitary bee species found on contemporary farmland in southern England, the majority of common species are polylectic and forage from a wide range of flowering plants. Whilst overall bee diversity was high, representing almost half the regional total (Baldock 2008), most of the generalist solitary bee species and almost all the specialised oligolectic solitary bee species were too scarce to allow formal diet characterisation. Within characterised bees there was a strong relationship between diet breadth and frequency of occurrence, with more generalist species found on a greater number of farms. Whilst earlier studies have shown that generalist bees are less sensitive to agricultural intensification (Bommarco et al. 2010; De Palma et al. 2015), the level of generalisation has not previously been shown to be a good predictor of frequency of occurrence. As more specialised bees are de facto less flexible in their dietary choices, the loss of floristic diversity resulting from agricultural intensification is likely to be the main driver behind their range declines over the past century (Scheper et al. 2014; Ollerton et al. 2014). Since bee species richness is strongly associated with plant species richness (Potts et al. 2003; Wood et al. 2015), the effectiveness of agri-environment schemes in providing resources for a wider variety of bee species is likely to be enhanced by increasing the number of flowering plant species in seed mixes (Scheper et al. 2015). For oligolectic species, only five out of 14 species were recorded collecting pollen from plant species currently sown as part of agri-environment schemes (Table 2). The addition of a wider range of species such as *H. radicata* to these mixes would provide resources for a wider variety of specialised bee species. However, increasing floristic diversity is not a straightforward process as sown species do not always develop or persist depending on the local soil type, the plant establishment method, competition between sown and unsown plants and subsequent management (Pywell et al. 2011). Many of these important plant species are associated with low intensity grassland and hedgerow habitats, so complementary techniques that maintain and improve floristic richness in long term habitats may also be effective.

Pollen load analysis provided a more complete description of solitary bee pollen diets than direct observation, consistently detecting pollens from a greater number of plant families across all bee species. In particular, this analysis identified plant families represented by woody plant genera whose flowers are often located well above the height of the surveyor. Due to this spatial structuring, these woody genera are consequently under-recorded as pollen sources by direct observations, and as a result their importance to bees may be widely underestimated. Beil et al. (2008) produced similar findings with a small number of bees collected on low growing herbaceous plants found to be carrying pollen from woody plant genera. In some cases in this study the nearest trees of this type were located over 1000 metres away from the collection point. There is a positive relationship between bee body size and foraging distance, with small bees predicted to have a maximum foraging distance of only a few hundred metres (Greenleaf et al. 2007). However, detailed study of experimental solitary bee populations confirms that whilst the majority of individuals do only forage over distances of a few hundred metres, a small proportion are able to successfully forage over 1000 m from their nest, even in small bees such as *Hylaeus* (Zurbuchen et al. 2010). Since at least a proportion of solitary bee individuals are capable of making long pollen foraging trips of over 1000 m, the importance of flowering plants that may not be immediately apparent in the sampling location and their contribution to the diet of farmland bees should be considered, further emphasising the importance of pollen load analysis for building a more complete picture of solitary bee diets.

Whilst host plant use for studied species broadly conformed to the literature, three polylectic species were characterised here as oligolectic. All analysed pollen loads of *Lasioglossum villosulum* were comprised of Asteraceae pollen, 98.2 % from the ‘hawkish’ Asteraceae genera *Hypochaeris* (49.3 %), *Leontodon* (34.8 %) and *Crepis* (14.0 %). This would clearly suggest broad oligolecty under the conditions laid out by Müller and Kuhlmann (2008), but *L. villosulum* is known to be at least occasionally polylectic, for example in the Mediterranean collecting pollen from *Ecballium elaterium*, a member of the Cucurbitaceae (Rust et al. 2003). At least in Britain, *L. villosulum* should probably be considered an oligolectic species, or a polylectic species with an extremely strong preference for Asteraceae. Both *A. alfkenella* and *A. minutuloides* were characterised as oligolectic on Apiaceae, though only summer generation females were collected. Both species are bivoltine in mainland Europe (Amiet et al. 2010), but in Britain it has been noted for a long time that both species are markedly less common in the spring generation (Perkins 1919; Baldock 2008). In the present study no spring generation males or females were recorded. Both species are considered polylectic as the spring generation forages from various plant families but the summer generation shows a strong preference for Apiaceae (Perkins 1919; Westrich 1989; Amiet et al. 2010). Because of the lack of spring females, neither species was considered to be oligolectic in this study.

The basal clades of most bee families include a high proportion of oligolecs (Westrich 1989; Wcislo and Cane 1996) and it has been argued that oligolecty is the basal state in bees with polylecty being a derived state with multiple origins (Müller 1996; Danforth et al. 2013). One of the suggested mechanisms by which oligolecty is maintained is that plants may chemically protect their pollen to prevent overexploitation, thus necessitating specialisation to process difficult metabolites (Praz et al. 2008). Asteraceae pollen is known to have a low protein content (Roulston et al. 2000; Hanley et al. 2008), is lacking in essential amino acids (Wille et al. 1985) and may possess a toxic pollenkitt, the oily liquid found on the surface of the pollen grain (Williams 2003). Consequently Asteraceae pollen is difficult to utilize by non-specialised bees, even in widely polylectic species such as the honey bee (Herbert et al. 1970) and solitary bees such as *Osmia lignaria* (Williams 2003). Even bees specialised on Asteraceae pollen may incur other costs such as extended development time, as in a comparison of specialised bees the Asteraceae oligolec *Heriades truncorum* had the longest development time on its preferred pollen despite being the smallest bee in the comparison (Praz et al. 2008). In reviewing host plant use and diet breadth in 60 species of Western Palearctic *Colletes*, Müller and Kuhlmann (2008) found that 12 species collected pollen exclusively from Asteraceae with a further two showing a strong preference. However, amongst widely polylectic species Asteraceae pollen played a very marginal role, with pollen loads from 27 species not containing Asteraceae pollen at all. This striking difference, suggesting a high degree of specialisation or almost total avoidance, is referred to as the Asteraceae paradox.

Interestingly, in the present study we found that four clearly polylectic solitary bee species collected a substantial proportion of their pollen from Asteraceae in the study area, these species being *Andrena flavipes* (56.0 %), *Lasioglossum calceatum* (46.9 %), *L. malachurum* (73.1 %) and *L. pauxillum* (62.1 %). The three *Lasioglossum* species also collected small amounts of *Ranunculus* pollen, a genus known to have pollen toxic to insects (Jürgen and Dötterl 2004). Additionally, after rarefaction, these four species had the widest diet of any of the characterised species and were each present on at least three-quarters of all surveyed farms. That these broad polylecs have developed the physiological mechanisms to digest a range of difficult pollens may be related to their long flight periods. *A. flavipes* is bivoltine, flying in the spring and again in the summer in discrete generations. *L. malachurum* and *L. pauxillum* are obligately primitively eusocial (Plateaux-Quénu 2008) and *L. calceatum* is facultatively eusocial with the eusocial phenotype dominating in the south of England (Davison and Field 2016). Producing two generations in a season, or in the case of social *Lasioglossum*, a worker and a reproductive generation, necessitates the ability to collect and digest pollen collected over a long flowering season from April to September. In contrast to these effectively bivoltine species, the Western Palearctic *Colletes* are almost always univoltine and the resultant shorter flight season and temporally limited resource competition may favour an all or nothing investment in the physiological capacity to process Asteraceae pollen. Indeed, xeric environments with short flowering seasons tend to have bee faunas with a greater degree of pollen specialisation (Minckley and Roulston 2006; Michener 2007). A more thorough understanding of the physiological mechanisms used by both specialised and generalist bees to detoxify and digest chemically protected pollen is necessary to better explain the different strategies pursued by foraging bees (Praz et al. 2008).

## Conclusions

The majority of solitary bees persisting on farmland in reasonable numbers are polylectic and make use of a wide variety of flowering plants. However, the level of generalisation is important, with the species with the widest diet breadth being found on the greatest number of farms. The ability to digest pollens from a large number of plant species is one reason that these more generalised bees are better able to deal with a wider variety of agricultural landscapes than bees with a narrower diet. Given that current agri-environment schemes targeted at pollinators do not result in an increase in either floristic or bee species richness at the farm scale (Wood et al. 2015), if the aim of agri-environment schemes is to support a diverse community of farmland bees this will require a change in scheme design to provide more appropriate foraging resources for more specialised bee species. This may rely on increasing the number of flowering plant species than are currently included in agri-environment schemes for pollinators.

## Electronic supplementary material

Below is the link to the electronic supplementary material.

Supplementary material 1 (XLSX 36 kb)

Supplementary material 2 (DOCX 12 kb)

Supplementary material 3 (DOCX 12 kb)

Supplementary material 4 (DOCX 13 kb)
