# Supplementary material for: Diet characterisation of solitary bees on farmland: dietary specialisation predicts rarity
Source: Biodivers Conserv. 2016 Aug 20;25(13):2655–71. doi: 10.1007/s10531-016-1191-x (PMC7175682; doi:10.1007/s10531-016-1191-x)
Supplement: Supplementary file 2 — Supplementary material 2 (DOCX 12 kb) [file 10531_2016_1191_MOESM2_ESM.docx]

APPENDIX II

Categories of bee host range as applied in this study. s.l. = sensu latu, s.s. = sensu strictu. From Müller and Kuhlmann (2008).

| Category | Subcategories | Definition |
| --- | --- | --- |
| Monolecty | - | Pollen collection on only one plant species even in the presence of one or more sympatric species of the same genus.^1^ |
| Oligolecty | Narrow oligolecty | Pollen collection from two to several species belonging to one plant genus. |
|  | Broad oligolecty | Pollen collection from two to several genera beloning to one plant tribe, subfamily or family. |
|  | Eclectic oligolecty | Pollen collection from two to four plant genera belonging to two or three plant families. |
| Polylecty s.l. | Polylectic with strong preference | Pollen collection from several plant families, but one plant clade (family, subfamiliy, tribe, genus or species) predominates. |
|  | Mesolecty | Pollen collection from more than four plant genera belonging to two or three plant families. |
|  | Polylecty s.s. | Pollen collection from various genera belonging to at least four plant families. |

^1^Pollen collection from one plant species in the absence of coflowering congenerics is referred to as a special case of narrow oligolecty
