# Supplementary material for: Diet characterisation of solitary bees on farmland: dietary specialisation predicts rarity
Source: Biodivers Conserv. 2016 Aug 20;25(13):2655–71. doi: 10.1007/s10531-016-1191-x (PMC7175682; doi:10.1007/s10531-016-1191-x)
Supplement: Supplementary file 3 — Supplementary material 3 (DOCX 12 kb) [file 10531_2016_1191_MOESM3_ESM.docx]

APPENDIX III

Inferring bee host ranges by microscopical analysis of scopal pollen loads of female bees based on two different methods: i) the number (or volume) of the pollen grains counted and ii) the individual composition of the pollen loads. From Müller and Kuhlmann (2008).

|  | Number (or volume) of pollen grains | Individual composition of pollen loads |
| --- | --- | --- |
| Monolecty | 95% or more of the pollen grains counted (or of the pollen grain volume) belong to one plant species. | 90% or more of females with pure loads of one plant species. |
| Narrow oligolecty | 95% or more of the pollen grains counted (or of the pollen grain volume) belong to one plant genus. | 90% or more of females with pure loads of one plant genus. |
| Broad oligolecty | 95% or more of the pollen grains counted (or of the pollen grain volume) belong to one plant tribe, subfamily or family. | 90% or more of females with pure loads of one plant tribe, subfamily or family. |
| Eclectic oligolecty | 95% or more of the pollen grains counted (or of the pollen grain volume) belong to the same two to four plant genera from two or three plant families.  Polylecty | Pollen grains of the same two to four plant genera from two or three families in 95% or more of the pollen loads. |
| Polylecty with a strong preference | 70–95% of the pollen grains counted (or of the pollen grain volume) belong to one plant clade (family, subfamily, tribe, genus or species). | 40–90% of females with pure loads of one plant clade (family, subfamily, tribe, genus or species) **AND** the preferred host plant clade in 75% or more of the pollen loads. |
| Mesolecty | 95% or more of the pollen grains counted (or of the pollen grain volume) belong to two or three plant families **AND** the most important clade contributes less than 70%. | Pollen grains of two or three plant families in 95% or more of the pollen loads. |
| Polylecty s.s. | Less than 70% of the pollen grains counted (or of the pollen grain volume) belong to one plant family **AND** the three most important plant families contribute less than 95%. | Less than 40% of females with pure loads of one plant family.  **OR**  Preferred host plant clade in less than 75% of the pollen loads AND pollen grains of the three most important plant families in less than 95% of the pollen loads. |
