# Supplementary material for: Diet characterisation of solitary bees on farmland: dietary specialisation predicts rarity
Source: Biodivers Conserv. 2016 Aug 20;25(13):2655–71. doi: 10.1007/s10531-016-1191-x (PMC7175682; doi:10.1007/s10531-016-1191-x)
Supplement: Supplementary file 4 — Supplementary material 4 (DOCX 13 kb) [file 10531_2016_1191_MOESM4_ESM.docx]

APPENDIX IV

Full list of bee species recorded during the study with species authorities

*Andrena angustior* Kirby 1802

*Andrena alfkenella* Perkins 1914

*Andrena bicolor* Fabricius 1775

*Andrena bucephala* Stephens 1846

*Andrena chrysosceles* Kirby 1802

*Andrena cineraria* Linnaeus 1758

*Andrena dorsata* Kirby 1802

*Andrena flavipes* Panzer 1799

*Andrena florea* Fabricius 1793

*Andrena fulva* Müller 1766

*Andrena fulvago* Christ 1791

*Andrena haemorrhoa* Fabricius 1781

*Andrena helvola* Linnaeus 1758

*Andrena humilis* Imhoff 1832

*Andrena labialis* Kirby 1802

*Andrena labiata* Fabricius 1781

*Andrena minutula* Kirby 1802

*Andrena minutuloides* Perkins 1914

*Andrena nigroaenea* Kirby 1802

*Andrena nitida* Müller 1776

*Andrena nitidiuscula* Schenck 1853

*Andrena scotica* Perkins 1916

*Andrena semilaevis* Perez 1903

*Andrena subopaca* Nylander 1848

*Andrena trimmerana* Kirby 1802

*Andrena wilkella* Kirby 1802

*Anthidium manicatum* Linnaeus 1758

*Anthophora furcata* Panzer 1798

*Anthophora plumipes* Pallas 1772

*Chelostoma campanularum* 1802

*Chelostoma florisomne* Linnaeus 1758

*Colletes daviesanus* Smith 1846

*Halictus rubicundus* Christ 1791

*Halictus tumulorum* Linnaeus 1758

*Hoplitis claviventris* Thomson 1872

*Hylaeus brevicornis* Nylander 1852

*Hylaeus communis* Nylander 1852

*Hylaeus confusus* Nylander 1852

*Hylaeus cornutus* Curtis 1831

*Hylaeus dilatatus* Kirby 1802

*Hylaeus hyalinatus* Smith 1842

*Hylaeus signatus* Panzer 1798

*Lasioglossum albipes* Fabricius 1781

*Lasioglossum calceatum* Scopoli, 1763

*Lasioglossum fulvicorne* Kirby 1802

*Lasioglossum laevigatum* Kirby 1802

*Lasioglossum lativentre* Schenck 1853

*Lasioglossum leucopus* Kirby 1802

*Lasioglossum leucozonium* Schrank 1781

*Lasioglossum malachurum* Kirby 1802

*Lasioglossum minutissimum* Kirby 1802

*Lasioglossum morio* Fabricius 1783

*Lasioglossum parvulum* Schenck 1853

*Lasioglossum pauperatum* Brule 1832

*Lasioglossum pauxillum* Schenck 1853

*Lasioglossum puncticolle* Morawitz 1872

*Lasioglossum smeathmanellum* Kirby 1802

*Lasioglossum villosulum* Kirby 1802

*Lasioglossum xanthopus* Kirby 1802

*Lasioglossum zonulum* Smith 1848

*Megachile centuncularis* Linnaeus 1758

*Megachile ligniseca* Kibry 1802

*Megachile versicolor* Smith 1844

*Megachile willughbiella* Kirby 1802

*Melitta leporina* Panzer 1799

*Melitta tricincta* Kirby 1802

*Osmia bicolor* Schrank 1781

*Osmia bicornis* Linnaeus 1758

*Osmia caerulescens* Linnaeus 1758

*Osmia leaiana* Kirby 1802

*Osmia spinulosa* Kirby 1802

*Panurgus calcaratus* Scopoli 1763
